# Prediction of Deoxypodophyllotoxin Disposition in Mouse, Rat, Monkey, and Dog by Physiologically Based Pharmacokinetic Model and the Extrapolation to Human

**DOI:** 10.3389/fphar.2016.00488

**Published:** 2016-12-16

**Authors:** Yang Chen, Kaijing Zhao, Fei Liu, Qiushi Xie, Zeyu Zhong, Mingxing Miao, Xiaodong Liu, Li Liu

**Affiliations:** Center of Pharmacokinetics and Metabolism, College of Pharmacy, China Pharmaceutical UniversityNanjing, China

**Keywords:** deoxypodophyllotoxin, physiologically based pharmacokinetic model, interspecies allometric scaling, unbound tissue-to-plasma concentration ratio, unbound fraction in plasma

## Abstract

Deoxypodophyllotoxin (DPT) is a potential anti-tumor candidate prior to its clinical phase. The aim of the study was to develop a physiologically based pharmacokinetic (PBPK) model consisting of 13 tissue compartments to predict DPT disposition in mouse, rat, monkey, and dog based on *in vitro* and *in silico* inputs. Since large interspecies difference was found in unbound fraction of DPT in plasma, we assumed that *K*_t:pl,u_ (unbound tissue-to-plasma concentration ratio) was identical across species. The predictions of our model were then validated by *in vivo* data of corresponding preclinical species, along with visual predictive checks. Reasonable matches were found between observed and predicted plasma concentrations and pharmacokinetic parameters in all four animal species. The prediction in the related seven tissues of mouse was also desirable. We also attempted to predict human pharmacokinetic profile by both the developed PBPK model and interspecies allometric scaling across mouse, rat and monkey, while dog was excluded from the scaling. The two approaches reached similar results. We hope the study will help in the efficacy and safety assessment of DPT in future clinical studies and provide a reference to the preclinical screening of similar compounds by PBPK model.

## Introduction

**FIGURE 1:**
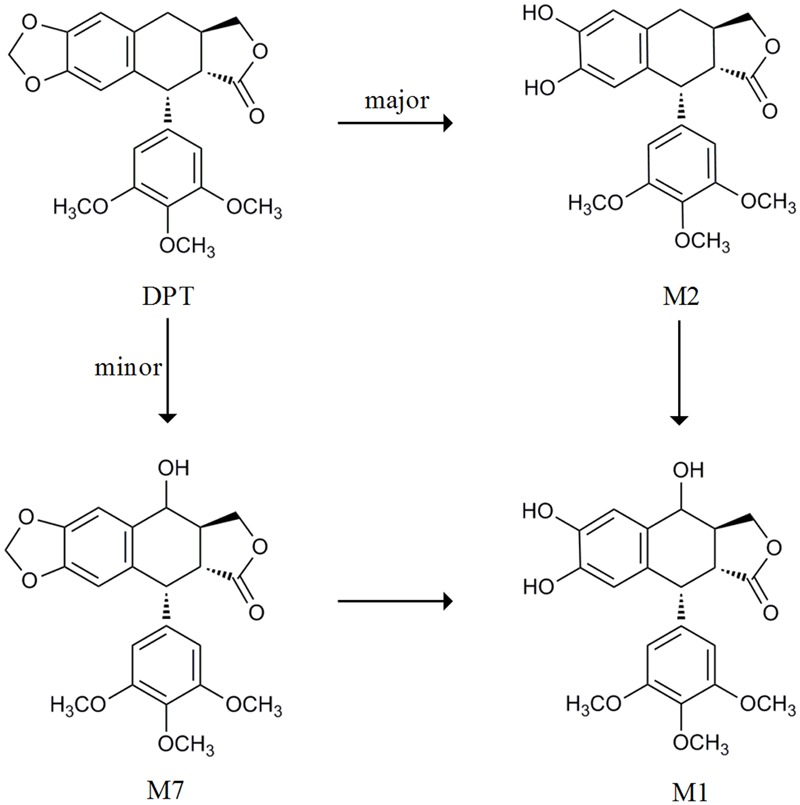
**The metabolic pathway from DPT to its main metabolites cited from Lee et al. (2008) and Xie et al. (2016)**.

Deoxypodophyllotoxin (**Figure 1**) is an active ingredient isolated from herbs like *Anthriscus sylvestris, Pulsatilla koreana*, and *Podophyllum emodi* (Wong et al., 2000; Khaled et al., 2013). Accumulating evidences have demonstrated that DPT possesses a variety of pharmacological activities, such as anti-tumor, anti-viral, anti-inflammatory, and anti-platelet aggregation effects (Gordaliza et al., 1994; Chen et al., 2000; Jin et al., 2008), among which anti-tumor effect is the most attractive. Its analogs etoposide and teniposide have already been widely used for the treatment of lung cancer, leukemia and lymphoma (Smith et al., 1994; Baldwin and Osheroff, 2005; Wu et al., 2014). DPT exerts its anti-tumor activity via affecting microtubule and modulating specific cell cycle-regulatory proteins (Khaled et al., 2013). As a promising anti-tumor candidate, DPT in an intravenous formulation of β-cyclodextrin inclusion complex (Zhu et al., 2010) has been developed and is undergoing its preclinical evaluation.

Our previous study reported that DPT was rapidly eliminated following intravenous administration to rats with a half-life about 80–100 min (Liu et al., 2016). Another study showed that DPT was transformed to seven metabolites in rat and human microsomes (Lee et al., 2008). We also quantitatively described the metabolism of DPT in hepatic microsomes of mouse, rat, monkey, dog, and human (Xie et al., 2016). Concluding by the metabolism studies above, DPT is mainly transformed to demethylenated metabolite M2 and mono-hydroxylated metabolite M7, both of which can be subsequently partly metabolized to mono-hydroxylated, demethylenated derivative M1 (**Figure 1**). M2 is major among all the metabolites. Our preliminary experiments proved that excretion of DPT in original form via urine and bile was less than 0.2% of the dose (4 mg/kg) following intravenous administration to rats, with high recoveries of M2 in the form of glucuronidated and sulfated conjugates. Nevertheless, no relevant *in vivo* report of DPT in other species was available to the best of our knowledge.

For a new candidate prior to its clinical phase, it is crucial and meaningful to gain a prediction of its pharmacokinetic profile in human based on the limited information from *in vitro* and animal studies. PBPK model and interspecies allometric scaling are two common methodologies for the prediction (Poulin et al., 2011; Vuppugalla et al., 2011). Although interspecies allometric scaling has been frequently used (Iavarone et al., 1999; Kang et al., 2009; Sayama et al., 2013), PBPK model, considered as a mechanism-based methodology, shows several advantages (Pang and Durk, 2010; Poulin et al., 2011; Jones and Rowland-Yeo, 2013). Interspecies allometric scaling highly depends on animal study, which should be performed with at least three animal species. However, the prediction with PBPK model may be accomplished only from *in vitro* and *in silico* data. PBPK model can also predict the dynamics of drug distribution in various tissues, leading to a better understanding of the relationship between target tissue exposure and drug safety/efficacy.

In this study we developed a whole-body PBPK model for the prediction of DPT disposition in four common preclinical species (mouse, rat, monkey, and dog), with parameters from *in vitro* and *in silico* study. After the validation by *in vivo* data and visual predictive checks in corresponding species, we attempted human pharmacokinetics projection by the model. Sensitivity analysis was conducted on the predicted pharmacokinetic profile in human by changing the metabolic velocity of DPT, volume of adipose tissue, unbound fraction in plasma of DPT and hepatic blood flow rate. Pharmacokinetic profile of DPT in human was also predicted by Dedrick plot based on animal data.

## Materials and Methods

### Chemicals and Reagents

The analytical standard of DPT (purity 99.88%), mono-hydroxylated metabolite M7 and β-cyclodextrin inclusion complex of DPT (content 3.36%) were kindly supplied by Medicinal and Chemical Institute of China Pharmaceutical University (Nanjing, China). Diazepam (purity 99.9%) used as internal standard was purchased from the National Institutes for Food and Drug Control (Beijing, China). Hepatic microsomes from mouse, rat, monkey, dog, and human were from the same source with those in our previous study (Xie et al., 2016). All the other reagents were of analytical grade and commercially available.

### Animals

Adult ICR mice, beagle dogs, and cynomolgus monkeys were purchased from Sino-British SIPPR/BK Lab Animal, Ltd. (Shanghai, China), Shanghai Jiagan Biotech, Co., Ltd (Shanghai, China) and Hainan New Source Biotech, Co., Ltd (Haikou, China), respectively. Animals were housed in constant room temperature and humidity on a normal 12 h light/dark cycle. Water and food were provided *ad libitum*. Animal experiments were carried out according to institutional guidelines for the care and the use of laboratory animals and approved by the Animal Ethics Committee of China Pharmaceutical University.

### Plasma Protein Binding Studies

Protein bindings of DPT in mouse, dog, and monkey plasma were evaluated in our study by a rapid equilibrium dialysis device (Linden Bioscience, Woburn, MA, USA) according to the manufacturer’s instruction. Protein binding values of DPT in rat and human plasma were obtained in a previous study by the same approach. Dialysis experiments were initiated by adding 300 μL of plasma containing DPT into donor cells and 500 μL of 100 mM phosphate buffer (pH 7.4) into receiver cells. After 6 h incubation at 37°C, the DPT concentrations in both cells were measured based on the LC–MS/MS method previously described (Liu et al., 2016). A preliminary study was conducted to determine the time to reach equilibrium state. Three initial DPT concentrations (0.5, 1.5, and 4.5 μg/mL) in rat and human plasma were selected to investigate whether non-linear binding occurred, while initial DPT concentration in plasma of mouse, dog, and monkey was set to be 1.0 μg/mL. All incubations were carried out in triplicate.

### Kinetics of M7 Formation from DPT in Hepatic Microsomes of Five Species

Kinetic parameters of M2, the major metabolite of DPT, were cited from our previous report (Xie et al., 2016). Here, the kinetics of M7 formation from DPT was depicted. Preliminary incubations were performed to optimize the conditions. The composition of microsomal incubation mixture and the procedure of incubation were the same with those in Xie et al. (2016), except that the incubation time was set to be 5 min for all the samples and microsomal protein levels were 0.02, 0.02, 0.05, 0.4, and 0.08 mg/mL for mouse, rat, monkey, dog, and human microsomes, respectively. A series of DPT concentrations from 0.0785 to 3.77 μM in microsomal incubation system (200 μL) were adopted. Incubations of each DPT concentration were carried out in triplicate. The formation of M7 was determined based on the LC–MS/MS assay described previously (Xie et al., 2016).

### *In vivo* Pharmacokinetic Study

Pharmacokinetic profiles of DPT in rats were cited from our previous study (Liu et al., 2016). In the present study, pharmacokinetics of DPT in mice, monkeys, and dogs were investigated following intravenous administration of DPT (β-cyclodextrin inclusion complex, dissolved in 0.9% normal saline within 2 h before administration). The dosages in the study were designed based on the pharmacological dose in tumor bearing mice and the toxicity in corresponding species. All experimental animals were fasted for 12 h prior to dosing.

#### Mice

In the mice study 132 ICR mice of about 5 weeks old (weighing about 20 g) were randomly divided into two groups and received 12.5 and 25.0 mg/kg of DPT via tail veins, respectively. Six mice (three males and three females) from each group were sacrificed at 2, 5, 10, 15, 30, 45, 60, 90, 120, 180, and 240 min post-dose and blood samples were collected into heparinized tubes. Heart, liver, lung, kidney, brain, muscle, and spleen were simultaneously collected at 5, 15, 60, and 120 min following i.v. administration (12.5 mg/kg of DPT) to mice. The tissue samples were weighed and homogenized in water (1:5, w/v).

#### Monkeys

Twelve cynomolgus monkeys of about 4 years old weighing 3.51 ± 0.85 kg were randomly divided into two groups by each of six (three males and three females). One group received 1.0 mg/kg of DPT via cephalic vein. Two doses of DPT (0.5 and 2.0 mg/kg) were successively given to another group via cephalic vein, separated by a 1-week washout interval. The blood samples were collected into heparinized tubes via another cephalic vein at pre-dose and 2, 5, 10, 20, 30, 45, 60, 90, 120, 180, and 240 min post-dose.

#### Dogs

Six beagle dogs of about 10 months old (three males and three females) weighing 8.65 ± 0.64 kg were subjected to 0.3 mg/kg of DPT via cephalic vein and blood samples were collected into heparinized tubes via another cephalic vein at pre-dose and 5, 15, 30, 60, 120, 180, 240, 300, 360, 420, 480, 600, 720, and 840 min post-dose.

All blood samples were centrifuged for 5 min at 8000 *g* for plasma. The plasma and tissue homogenate samples were stored at -70°C until analysis. DPT concentrations in plasma and tissue homogenates were measured by the LC–MS/MS method previously described (Liu et al., 2016).

Pharmacokinetic parameters were estimated using non-compartmental analysis by Phoenix WinNonlin software (version 6.4, Certara, Co., Princeton, NJ, USA). The estimated main pharmacokinetic parameters included AUC_0-tn_, AUC_0-∞_, *CL, t*_1/2_, MRT, and *V*_ss_.

### PBPK Model Development

**FIGURE 2:**
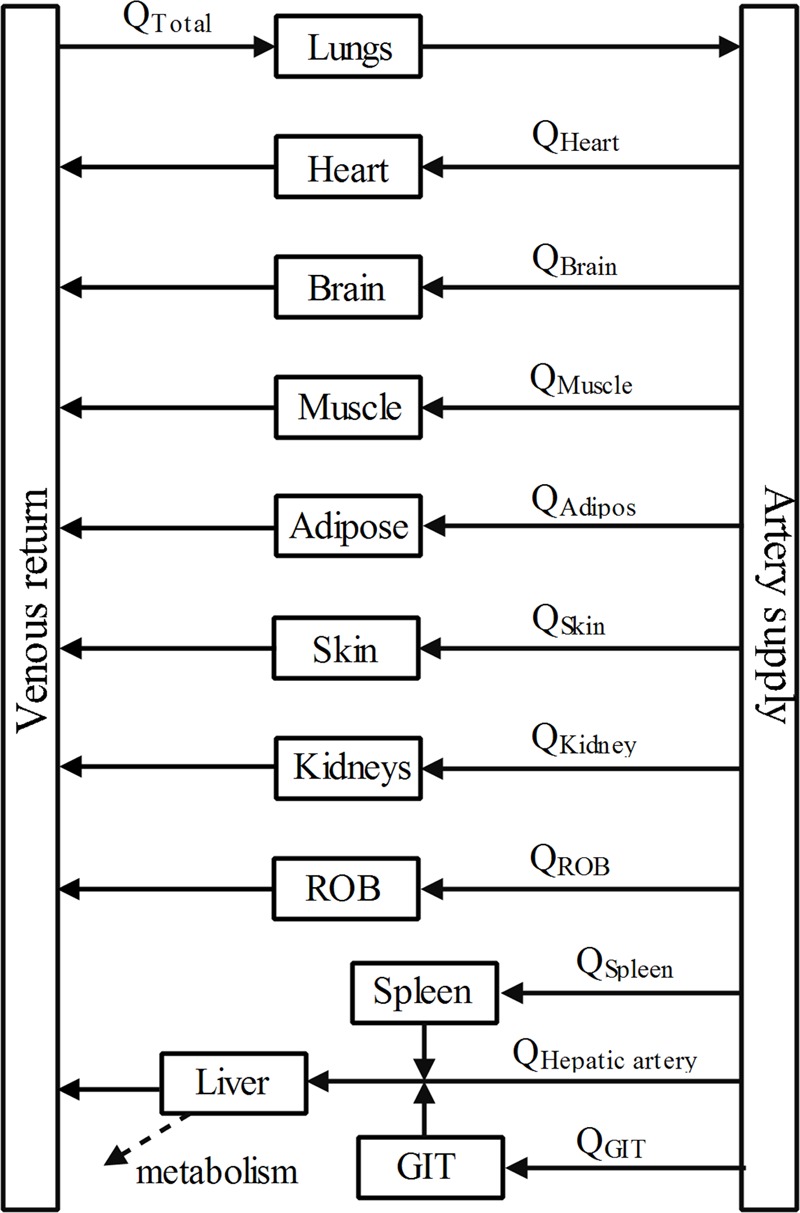
**Schematic diagram of whole-body PBPK model of DPT.** Arrows connecting compartments stand for the blood flows. GIT represents gastrointestinal tract and ROB represents the rest part of body.

A PBPK model (**Figure 2**) was constructed to describe the pharmacokinetic profiles of DPT in five species (mouse, rat, monkey, dog, and human). The essential structure of the model consisted of lung, heart, spleen, gastrointestinal tract, liver, kidney, brain, adipose, muscle, skin, arterial and venous blood, and rest of body. Disposition of DPT in tissues was illustrated by perfusion-rate limited model. It was assumed that DPT was mainly eliminated in liver via M2 and M7 formation in all five species. The differential equations were described as follows:

For non-elimination tissue compartments,

$$V_{\text{t}}\frac{dC_{\text{t}}}{dt}=Q_{\text{t}}\times (C_{\text{art}}-\frac{C_{\text{t}}}{K_{\text{t}:\text{pl}}/R_{\text{bp}}})$$

where *C*_t_, *V*_t_, *Q*_t,_ and *K*_t:pl_ represent the concentration of DPT, the volume, the blood flow rate and tissue-to-plasma concentration ratio of corresponding tissue compartments. *C*_art_ means the drug concentration in arterial blood compartment. *R*_bp_ means blood-to-plasma concentration ratio, which was assumed to be unity in the study, according to the reasons provided in Poulin and Theil (2002).

For arterial blood compartment (subscript *art*),

$$V_{\text{art}}\frac{dC_{\text{art}}}{dt}=Q_{\text{total}}\times (\frac{C_{\text{lun}}}{K_{\text{lun}:\text{pl}}/R_{\text{bp}}}-C_{\text{art}})$$

For venous blood compartment (subscript *ven*),

$$V_{\text{ven}}\frac{dC_{\text{ven}}}{dt}=\sum_{t}\left(Q_{\text{t}}\times \frac{C_{t}}{K_{\text{t}:\text{pl}}/R_{\text{bp}}}\right)-Q_{\text{total}}\times C_{\text{ven}}$$

For lung compartment (subscript *lun*),

$$V_{\text{lun}}\frac{dC_{\text{lun}}}{dt}=Q_{\text{total}}\times (C_{\text{ven}}-\frac{C_{\text{lun}}}{K_{\text{lun}:\text{pl}}/R_{\text{bp}}})$$

where *Q*_total_ represents the cardiac output.

And for liver compartment,

$$\begin{array}{c} V_{\text{liv}}\frac{dC_{\text{liv}}}{dt}=Q_{\text{hep}}\times C_{\text{art}}+Q_{\text{gut}}\times \frac{C_{\text{gut}}}{K_{\text{gut}:\text{pl}}/R_{\text{bp}}}+Q_{\text{spl}}\times \;\\ \frac{C_{\text{spl}}}{K_{\text{spl}:\text{pl}}/R_{\text{bp}}}-(Q_{\text{hep}}+Q_{\text{gut}}+Q_{\text{spl}})\times \;\\ \frac{C_{\text{liv}}}{K_{\text{liv}:\text{pl}}/R_{\text{bp}}}-PBSF\times (CL_{\text{M2}}+CL_{\text{M7}}) \end{array}$$

where subscripts *liv, gut and spl* refer to liver, gastrointestinal tract and spleen, respectively. *Q*_hep_ represents blood flow rate of hepatic artery. *PBSF* is the amount of total hepatic microsomal protein, which equals to the product of the microsomal protein yield and liver weight. *CL*_M2_ and *CL*_M7_ denote the clearance via the formation of metabolite M2 and M7, respectively.

*K*_t:pl_ of DPT in rat were estimated according to a method previously described based on tissue composition of rat (Ruark et al., 2014). Ionization, lipophilicity and the fraction unbound in plasma of compound were incorporated into the calculation. As a neutral compound, DPT was considered to be unionized in physiological environment and the logarithm of octanol-water partition coefficient (Log P) of DPT was calculated to be 2.68 by Chemdraw 15.1 (PerkinElmer Informatics, Inc., Waltham, MA, USA).

Coding and solving of the PBPK model were conducted by Phoenix WinNonlin software (version 6.4, Certara, Co., Princeton, NJ, USA). Pharmacokinetic profiles of DPT in plasma of four animal species and in tissues of mouse were predicted. Pharmacokinetic parameters of the predicted profiles were estimated using non-compartmental analysis. The predictions were compared with corresponding sets of *in vivo* observations. The accuracy of predicted values was assessed by fold-error defined as follows:

If the observed value was greater than the predicted value, fold-error was observed value divided by predicted value;
If the observed value was less than the predicted value, fold-error was predicted value divided by observed value.

The fold-error less than two denoted a successful prediction. The validated PBPK model was subsequently applied to predict pharmacokinetic profile in human.

### Visual Predictive Checks of the Model

To validate the model in animal populations, we assumed that inter-individual variability existed in hepatic blood flow rate and metabolic velocity of DPT. We introduced inter-individual variability by exponential model and intra-individual variability by multiplicative residual error model, to simulate the pharmacokinetic profiles in animal populations. The first order conditional estimation of Lindstrom-Bates (FOCE L-B) method was used in the simulation. All values of parameters listed in **Tables 1** and **2** as well as unbound fraction in plasma of DPT were regarded as typical values and fixed during the simulation, while the variances of hepatic blood flow rate and metabolic velocity (regarded as random effect parameters) together with standard deviation of intra-individual error were estimated. The constructed population models were subsequently subjected to visual predictive checks based on 1000 times of simulations. The 5, 50, and 95th percentiles of the simulations were plotted along with the observed data for visual inspection. Simulation and validation of the population models were performed on Phoenix NLME module (version 1.3, Certara, Co., Princeton, NJ, USA).

**Table 1:** Physiological parameters of five species used in PBPK model.

	Rat (0.25 kg)			Mouse (0.02 kg)			Monkey (4 kg)			Dog (8.5 kg)			Human (70 kg)		
	Volume*^a^* (mL)	Blood flow*^a^* (mL/min)	*K*_t:pl_	Volume*^b^* (mL)	Blood flow*^c^* (mL/min)	*K*_t:pl_	Volume*^d^* (mL)	Blood flow*^e^* (mL/min)	*K*_t:pl_	Volume*^f^* (mL)	Blood flow*^f^* (mL/min)	*K*_t:pl_	Volume*^g^* (L)	Blood flow*^g^* (L/min)	*K*_t:pl_
Adipose	19.00	5.82	21.63	1.73	0.72	11.36	325.89	20.56	3.25	1500.00	50.00	0.64	10.00	0.26	26.33
Liver*^h^*	9.15	14.53	1.68	1.10	1.94	0.88	108.00	144.79	0.25	213.00	323.33	0.05	1.69	1.48	2.05
Muscle	101.00	23.10	0.75	7.67	0.91	0.40	2000.00	227.02	0.11	4250.00	170.00	0.02	35.00	0.75	0.92
Lungs	1.25	83.90	1.72	0.15	8.00	0.90	26.40	893.78	0.26	85.00	968.33	0.05	1.17	5.60	2.09
Kidneys	1.83	11.71	1.41	0.33	1.30	0.74	24.00	140.32	0.21	97.00	170.00	0.04	0.28	1.24	1.72
Brain*^i^*	1.43	1.66	2.77	0.33	0.26	1.46	72.00	48.26	0.42	50.00	145.00	0.08	1.45	0.70	3.37
Heart	0.83	4.07	0.97	0.10	0.28	0.51	13.60	43.80	0.15	43.00	43.33	0.03	0.31	0.24	1.18
Spleen	0.50	1.66	1.06	0.07	0.09	0.55	6.40	22.34	0.16	22.00	13.33	0.03	0.19	0.08	1.29
Skin	47.50	4.82	1.42	3.30	0.41	0.75	400.00	69.71	0.21	364.00	18.33	0.04	7.80	0.30	1.73
Gastrointestinal tract	6.75	10.88	1.00	0.85	1.50	0.53	184.00	79.55	0.15	228.00	265.00	0.03	1.65	1.10	1.22
Rest of body*^j^*	40.38	17.36	0.0100	3.39	2.18	0.0052	546.11	199.31	0.0015	1223.00	48.33	0.0003	5.26	0.63	0.0122
Vein*^k^*	13.60	∖	∖	0.65	∖	∖	195.73	∖	∖	284.00	∖	∖	3.47		∖
Artery*^k^*	6.80	∖	∖	0.33	∖	∖	97.87	∖	∖	141.00	∖	∖	1.73		∖
*PS* (mL/min)		0.0133			0.0024			0.0849			0.1407			0.5780	

*^a^Values were from Luttringer et al. (2003).*

*^b^Values were from Bi et al. (2016). Data of 30 g mouse were changed to 20 g mouse by assumption that organ volume was proportional to body weight.*

*^c^Values were from Davies and Morris (1993), except that the blood flow rate of adipose and brain were from Lyons et al. (2013).*

*^d^Values were from Davies and Morris (1993). Data of 5 kg monkey were changed to 4 kg monkey by assumption that organ volume was proportional to body weight, except that the volume of adipose was from Brown et al. (1997).*

*^e^The cardiac output and the blood flow distribution of organs were from Hoffbrand and Forsyth (1969) and Fisher et al. (2011), respectively.*

*^f^Values were from Kawai et al. (1994).*

*^g^Values were from Davies and Morris (1993).*

*^h^The blood flow rate of liver was assumed to be the sum of blood flow rates of hepatic artery, gastrointestinal tract, and spleen.*

*^i^The vascular and extravascular volumes of brain were assumed to account for 3 and 97% of the total brain volume, respectively, according to mouse data in Hu et al. (2014).*

*^j^The volume of rest of body equaled to total body volume subtracted by the summed volume of listed organs. The blood flow rate of rest of body equaled to the cardiac output subtracted by the summed blood flow rate of listed organs. *K*_t:pl_ value of rest of body in rat was assumed to be 0.01, for the negligible distribution in the rest of body (mainly composed of bones).*

*^k^The volume of artery and vein were assumed to account for 1/3 and 2/3 of total volume of blood, respectively, if the data was unavailable.*

**Table 2:** Metabolic parameters of five species used in PBPK model.

	Mouse	Rat	Monkey	Dog	Human
*PBSF^∗^*(mg protein/body)	49.28	409.92	5270.40	16592.70	82472.00
*K*_m1,M2_ (μM)	1.81	0.05	0.08	0.09	0.24
*V*_max1,M2_ (nmol/min/mg protein)	3.43	4.93	0.40	0.03	0.32
*K*_m2,M2_ (μM)	n	n	2.26	9.47	n
*V*_max2,M2_ (nmol/min/mg protein)	n	n	1.02	0.10	n
*K*_m,M7_ (μM)	1.21	1.27	0.31	0.52	1.53
*V*_max,M7_ (pmol/min/mg protein)	42.76	150.03	31.86	2.64	24.38
γ	1.55	1.55	1.78	1.45	1.22

*^∗^The microsomal protein yields in mouse, rat, monkey, dog, and human were 44.8, 44.8, 48.8, 77.9, and 48.8 mg protein/g liver, respectively, according to Naritomi et al. (2001). The data of mouse and monkey were assumed to be equal to those of rat and human, respectively. PBSF was the product of the microsomal protein yield and the liver weight in **Table 1**.*

### Sensitivity Analysis

The previous study showed M2 formation from DPT in human microsomes was mainly mediated by CYP2C9 and CYP2C19 (Xie et al., 2016), which were reported to exhibit marked inter-individual variability in their expression and catalytic activity (Hirota et al., 2013). In addition, DPT possessed high adipose-to-plasma concentration ratio and high plasma protein binding. Therefore, the sensitivity analysis was conducted to evaluate the impacts of alterations in metabolic velocity of DPT, adipose volume, unbound fraction of DPT in plasma and hepatic blood flow rate on pharmacokinetic behavior of DPT in human. The variation was set to be 10-fold for metabolic velocity and twofold for hepatic blood flow rate, respectively. Variations for both adipose volume and unbound fraction in plasma were designed to be threefold.

### Interspecies Scaling by Allometric Equation

We also tried to predict the pharmacokinetics of DPT in human basing on the pharmacokinetics in animal species by allometric scaling method (Dedrick et al., 1970; Boxenbaum and Ronfeld, 1983). *V*_ss_ and *CL* of DPT across species were expressed as follows:

$$V_{\text{ss}}=\;\alpha_{1}W^{\beta_{\text{1}}}\text{and}CL=\;\alpha_{2}W^{\;\beta_{2}}$$

where *W* is body weight of the corresponding species, *α* and *β* are the coefficient and exponent, respectively. *α* and β values were estimated following logarithmic / logarithmic transformation [*lg*(*V*_ss_) *= lg*(α_1_)*+*β_1_*lg*(*W*) and *lg*(*CL*) *= lg*(α_2_)*+*β_2_*lg*(*W*)] and unweighted least-squares regression analysis by IBM SPSS software (version 19.0, IBM, Co., Armonk, NY, USA). The plasma concentration(*C*)-time(*t*) data of DPT from different species were graphed using Dedrick plot, in which *X*-axis was physiological time *t′*(*t′ = t/W*^β1-β2^) and *Y*-axis was normalized concentration *C′*[*C′ = C/(D/W*^β1^*)*], where *D* represents the dose. The normalized concentration-physiological time data were fitted to two-compartment model by Phoenix WinNonlin software (version 6.4, Certara, Co., Princeton, NJ, USA). The plasma concentration-time profile of DPT in 70 kg human was then obtained via reverse transformation from the Dedrick plot. The predicted concentration-time profile and the estimated pharmacokinetic parameters in human were compared with those by PBPK model.

## Results

### Plasma Protein Binding of DPT in Five Species

Human plasma protein binding values of DPT at 0.5, 1.5, and 4.5 μg/mL were 93.13 ± 0.60, 93.31 ± 0.76, and 93.88 ± 0.46%, respectively. Rat plasma protein binding values of DPT at 0.5, 1.5, and 4.5 μg/mL were 94.39 ± 0.75, 94.52 ± 0.50, and 94.91 ± 0.33%, respectively. These results proved that DPT possessed high affinity to plasma protein and the binding was linear within the tested concentrations. The average protein binding values of DPT at the tested three concentrations in human and rat plasma were 93.44 and 94.61%, respectively. Protein binding values of DPT in mouse, monkey and dog plasma were 97.17 ± 0.25, 99.19 ± 0.03, and 99.84 ± 0.03%, respectively. Notable interspecies difference was observed in unbound fraction of DPT in plasma, which varied from 6.56% in human to 0.16% in dog.

### PBPK Model Development and Validation

PBPK model was developed for predicting pharmacokinetic profiles of DPT in rats first and then extrapolated to mouse, dog, and monkey. *K*_t:pl_ values in tissues of rat were calculated according to the method previously described (Ruark et al., 2014). A high *K*_t:pl_ was obtained in adipose tissue, indicating that DPT possessed high affinity to adipose tissue. *K*_gut:pl_ was assumed to be one in rat due to the unavailable tissue composition of gastrointestinal tract. Given the large interspecies difference in unbound fraction of DPT in plasma, unbound tissue-to-plasma concentration ratio (*K*_t:pl,u_) was incorporated in our PBPK model. *K*_t:pl,u_ is the ratio of the concentration in tissue to the unbound concentration in plasma, which was assumed identical across species (Rodgers et al., 2005). Thus, *K*_t:pl_ values in corresponding species could be derived from those of rat (*K*_t:pl,rat_) based on the equation *K*_t:pl_ = *K*_t:pl,rat_ ×*f*_up_ */f*_up,rat_, where *f*_up_ and *f*_up,rat_ are unbound fractions of DPT in plasma of the corresponding species and rat, respectively.

Preliminary prediction of DPT concentrations in brain of mice demonstrated a significant overestimation using the perfusion-rate limited model, therefore brain model was refined to be a permeability-limited model as follows:

$$\begin{array}{c} V_{1}\frac{dC_{1}}{dt}=Q_{\text{bra}}(C_{\text{art}}-C_{1})-PS(C_{1}-C_{2}/K_{\text{bra}:\text{pl}})\\ and\\ V_{2}\frac{dC_{2}}{dt}=PS(C_{1}-C_{2}/K_{\text{bra}:\text{pl}}) \end{array}$$

where *C* and *V* are the concentration of DPT and compartment volume, respectively. Subscript 1 and 2 denote vascular and extravascular compartment, respectively. *Q*_bra_ and *K*_bra:pl_ represent the blood flow rate of brain and brain-to-plasma concentration ratio, respectively. *PS* is the permeability-surface product of DPT in brain and the value of mouse (*PSmouse*) was estimated by fitting the model to brain concentration data. The PS values in brain of other species (*PS*_i_) were then scaled from data of mouse by the following equation (Hu et al., 2014):

$$PS_{\text{i}}=PS_{\text{mouse}}\times {\left(\frac{W_{\text{i}}}{W_{\text{mouse}}}\right)}^{0.67}$$

where *W*_i_ and *W*_mouse_ are the body weights of the corresponding species and mouse, respectively.

*CL*_M2_ and *CL*_M7_ were obtained from the previous and the present study, respectively. For the clearance via M2 formation:

$$CL_{\text{M2}}=\sum \frac{V_{\max,\text{i},\text{M2}}\times f_{\text{up}}\times C_{\text{liv}}/K_{\text{liv}:\text{pl}}}{K_{\text{m},\text{i},\text{M2}}+f_{\text{up}}\times C_{\text{liv}}/K_{\text{liv}:\text{pl}}}$$

where *K*_m,i,M2_, and *V*_max,i,M2_ are kinetic parameters of the metabolism from DPT to M2 in hepatic microsomes and were cited from our previous report (Xie et al., 2016).

**FIGURE 3:**
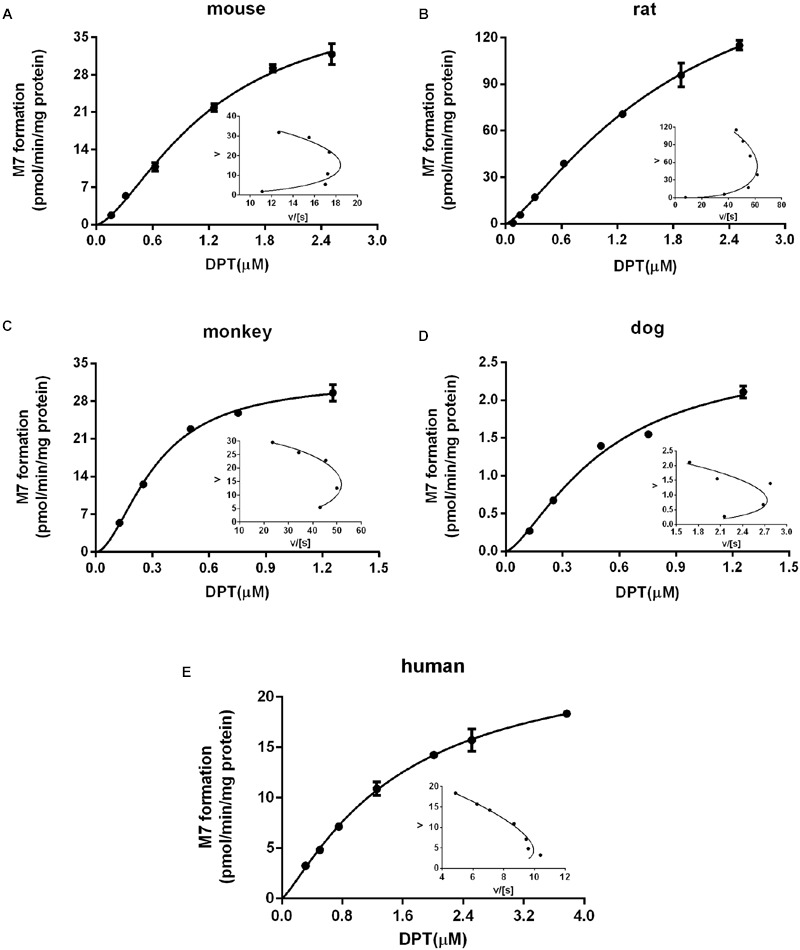
**Kinetic profiles of M7 formation in liver microsomes from (A)** mouse, **(B)** rat, **(C)** monkey, **(D)** dog, and **(E)** human. The insets are Eadie–Hofstee plots of the kinetic profiles. Dots and error bars represent the mean value and standard deviation of observed data at each DPT concentration, respectively (*n* = 3). Lines denote the simulated kinetic curves.

For the clearance via M7 formation, since the kinetics exhibited auto-activation features (**Figure 3**), a Hill equation was used to characterize the kinetics:

$$CL_{\text{M7}}=\frac{V_{\text{max},\text{M7}}\times {\left(f_{\text{up}}\times C_{\text{liv}}/K_{\text{liv}:\text{pl}}\right)}^{\gamma }}{K_{\text{m},\text{M7}}^{\gamma }+{\left(f_{\text{up}}\times C_{\text{liv}}/K_{\text{liv}:\text{pl}}\right)}^{\gamma }}$$

where *K*_m,M7_, *V*_max,M7_ and γ are kinetic parameters and Hill coefficient estimated from the kinetics of M7 formation in the present study.

**FIGURE 4:**
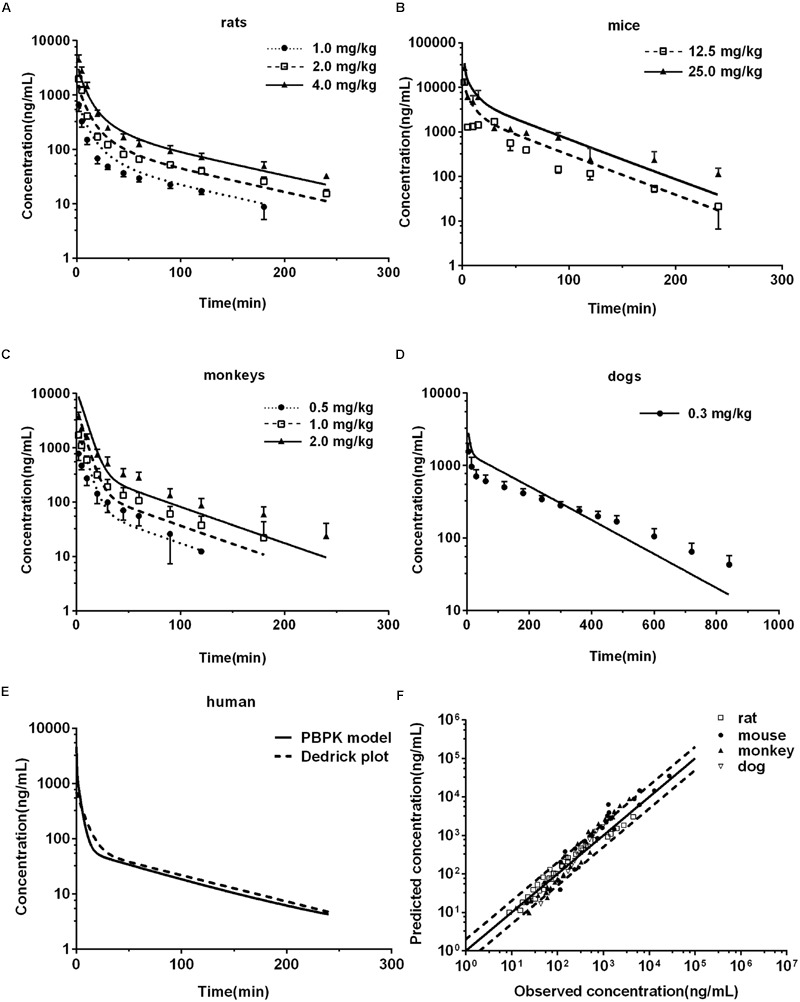
**Observed and predicted plasma concentration-time profiles of DPT in rats (A)** (*n* = 10), mice **(B)** (*n* = 6), monkeys **(C)** (*n* = 6), and dogs **(D)** (*n* = 6). Dots and error bars represent the mean value and standard deviation of observed data at each time point, respectively. Lines denote the predicted plasma concentration-time profiles. **(E)** Compares the human plasma concentration-time profiles of DPT predicted by Dedrick plot and PBPK model. **(F)** Represents the relationship of mean observed and predicted plasma concentration of DPT at each time point in four animal species, in which solid and dashed lines indicate unity and twofold errors between predicted and observed data, respectively.

All parameters used in the PBPK model were listed in **Tables 1** and **2**. The predictions were validated by *in vivo* pharmacokinetic data (**Figures 4A–D,F**).

#### Rats

Pharmacokinetic profiles of DPT in rat following intravenous administration (1.0, 2.0, and 4.0 mg/kg) were predicted by the developed PBPK model (**Figure 4A**) and corresponding pharmacokinetic parameters were obtained (**Table 3**). As seen in **Figure 4A** and **Table 3**, the predicted pharmacokinetic profiles and parameters were in line with the observations in our previous report (Liu et al., 2016). Fold-errors of all concentration data and parameters were less than two, indicating that the prediction was successful in rat. It was also found that DPT possessed high plasma clearance (close to the rate of hepatic blood flow) in rats, accompanied by a terminal half-life of 80–100 min and a volume of distribution above threefold body volume.

**Table 3:** The comparison of observed and predicted plasma pharmacokinetic parameters in five species.

	Dose	AUC_0-tn_		AUC_0-∞_		*CL*		*V*_ss_		MRT		*t*_1/2_	
	(mg/kg)	(μg⋅min/mL)		(μg⋅min/mL)		(mL/min/kg)		(L/kg)		(min)		(min)	
		Observed	Predicted	Observed	Predicted	Observed	Predicted	Observed	Predicted	Observed	Predicted	Observed	Predicted
Mouse	12.5	175.14	213.85	176.65	214.69	70.76	58.22	1.71	1.66	24.19	28.58	48.98	33.47
	25.0	359.39	486.31	369.10	488.18	67.73	51.21	2.54	1.44	37.52	28.05	58.82	33.45
Rat	1.0	9.55 ± 1.69	12.62	10.59 ± 1.74	13.63	96.98 ± 17.46	73.38	6.07 ± 1.39	3.68	63.27 ± 14.00	50.10	80.20 ± 19.11	69.75
	2.0	27.20 ± 2.93	26.16	29.23 ± 3.46	27.30	69.28 ± 8.18	73.27	4.29 ± 0.45	3.70	61.42 ± 8.18	50.51	84.42 ± 17.98	70.59
	4.0	62.45 ± 9.64	52.34	67.66 ± 9.33	54.62	60.17 ± 8.49	73.23	3.80 ± 1.02	3.70	62.40 ± 9.75	50.50	97.66 ± 14.93	70.59
Monkey	0.5	12.80 ± 3.41	21.05	13.94 ± 3.56	21.88	37.98 ± 10.07	22.85	1.19 ± 0.30	0.46	32.90 ± 11.68	20.28	32.71 ± 7.88	44.62
	1.0	29.98 ± 8.02	45.60	31.15 ± 8.23	46.29	33.84 ± 8.10	21.60	1.28 ± 0.24	0.44	39.58 ± 11.17	20.43	45.73 ± 10.85	44.79
	2.0	72.76 ± 11.72	102.19	75.23 ± 12.46	102.81	27.25 ± 4.85	19.45	1.22 ± 0.24	0.40	46.26 ± 13.40	20.72	57.52 ± 16.35	44.91
Dog	0.3	228.96 ± 35.71	295.24	240.84 ± 36.93	298.30	1.27 ± 0.17	1.01	0.35 ± 0.08	0.18	276.59 ± 45.23	175.52	186.77 ± 23.61	128.94
Human	16.0*^a^*	n	11.07	n	11.50	n	1.39*^b^*		61.94*^c^*		44.52	n	70.22

*^a^For human the unit of dose is mg.*

*^b^For human the unit of CL is L/min.*

*^c^For human the unit of *V*_ss_ is L.*

#### Mice

The developed PBPK model in rat was extrapolated to mouse. Plasma concentration-time profiles of DPT following intravenous administration (12.5 and 25.0 mg/kg) were predicted (**Figure 4B**) and corresponding pharmacokinetic parameters were estimated (**Table 3**). The predictions were further compared with *in vivo* data (**Figure 4B**; **Table 3**). *In vivo* results showed that the AUC values were proportional to dose levels. *CL* values were estimated to be 70.76 and 67.73 mL/min/kg for 12.5 and 25.0 mg/kg, respectively, suggesting that clearance of DPT in mice was in a dose-independent manner at the tested dose levels. It was also found that DPT exhibited a moderate plasma clearance in mice (about 70% of hepatic blood flow), with an associated *t*_1/2_ of about 40–60 min and a *V*_ss_ of about double of the body volume. The predicted pharmacokinetic profiles were comparable to the observed profiles, and the fold-errors of all the predictive parameters were less than two, implying a successful prediction.

**FIGURE 5:**
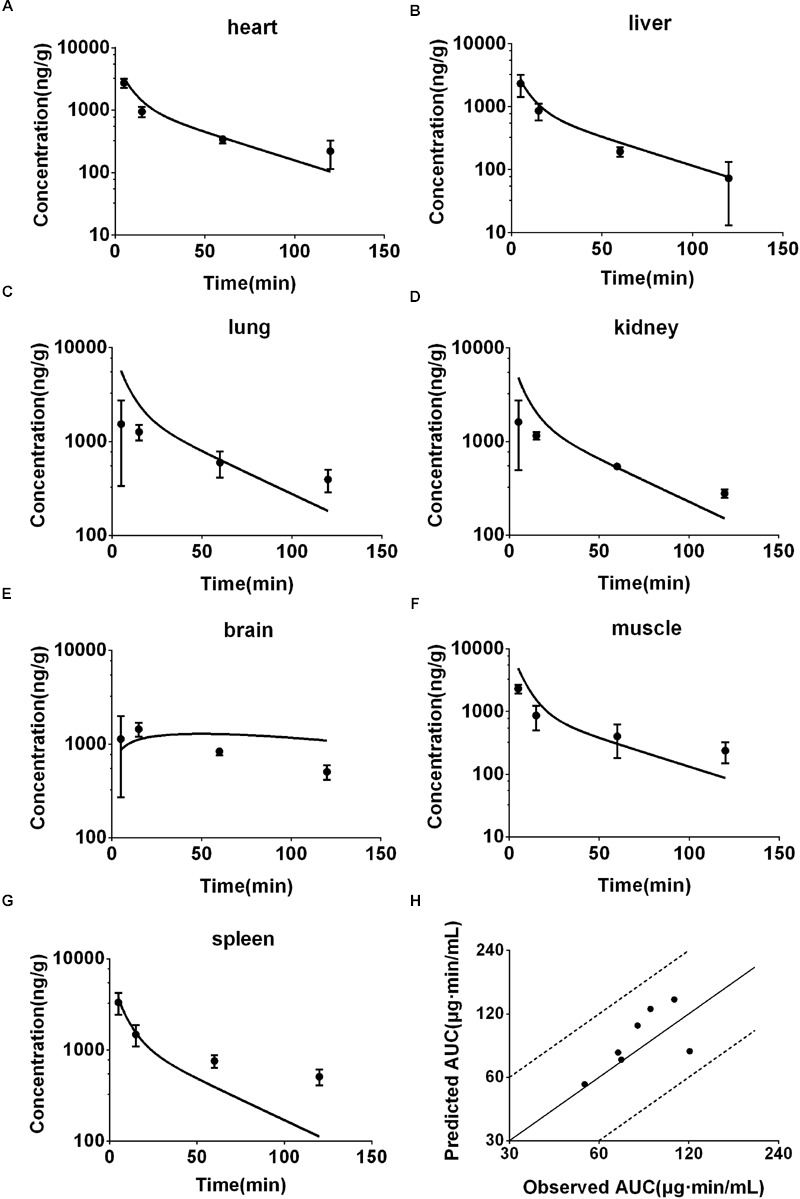
**Observed and predicted concentration-time profiles of DPT in heart (A)**, liver **(B)**, lung **(C)**, kidney **(D)**, brain **(E)**, muscle **(F)**, and spleen **(G)** of mice (12.5 mg/kg, *n* = 6). Dots and error bars represent the mean value and standard deviation of observed data at each time point, respectively. Lines represent the predicted concentration-time profiles. **(H)** Represents the relationship of observed and predicted AUC_0-120__min_ of DPT in the tissues above, in which solid and dashed lines indicate unity and twofold errors between predicted and observed data, respectively.

Concentrations of DPT in heart, liver, lung, muscle, brain, kidney, and spleen of mouse following intravenous injection (12.5 mg/kg) were simultaneously predicted and the prediction was validated by the actual observations at 5, 15, 60, and 120 min following dosing (**Figures 5A–G**). The exposures (AUC_0-120__min_) from the predicted profiles in the related tissues of mouse were in good agreement with the *in vivo* values, with fold-errors less than two (**Figure 5H**).

#### Monkeys

The developed PBPK model in rat was then extrapolated to monkey. The plasma concentration-time curve of DPT in monkeys following intravenous administration of 0.5, 1.0, and 2.0 mg/kg were predicted (**Figure 4C**) and the pharmacokinetic parameters were estimated (**Table 3**). *In vivo* pharmacokinetics indicated that plasma clearance of DPT in monkeys trended downward when the dose level increased, but no significant difference was observed. In monkeys, DPT exhibited a moderate to high *CL* (66–90% of hepatic blood flow), with a *t*_1/2_ ranging from 32 to 58 min and a *V*_ss_ slightly higher than total body volume. The predicted pharmacokinetic profiles by PBPK model were consistent with the *in vivo* data. The pharmacokinetic parameters estimated from the predicted profiles were within twofold error of the observed data except *V*_ss_ and MRT. The results demonstrated that the prediction in monkey was desirable.

#### Dogs

The developed PBPK model in rat was further extrapolated to dog. Plasma concentration-time data of DPT (i.v. 0.3 mg/kg) were predicted and further validated by *in vivo* data (**Figure 4D**; **Table 3**). The observed results revealed that the disposition of DPT in dogs was greatly different from the other three species. In dogs, DPT exhibited a very low *CL* (only 3.4% of hepatic blood flow), a long *t*_1/2_ of 187 min and a low *V*_ss_ (0.35 L/kg). However, the developed PBPK model still gave an appropriate prediction for pharmacokinetic profile of DPT in dogs. All the predicted pharmacokinetic parameters were within twofold error of the observed values.

### Visual Predictive Checks of the Model

**FIGURE 6:**
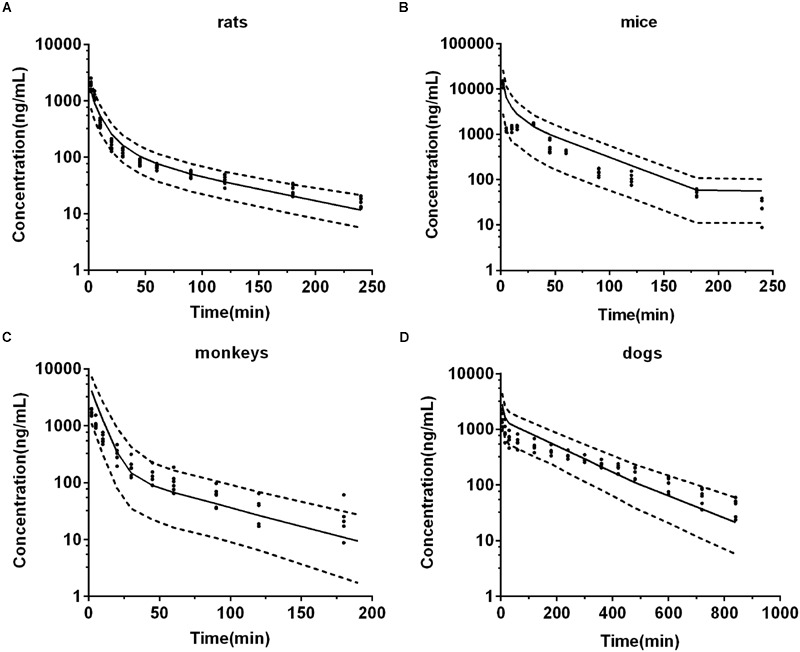
**The visual predictive checks of predicted plasma concentration-time profiles of DPT in 2.0 mg/kg rats (A)**, 12.5 mg/kg mice **(B)**, 1.0 mg/kg monkeys **(C),** and 0.3 mg/kg dogs **(D)**. Dots represent the observed individual data. Solid lines represent the 50th percentiles and dashed lines represent the 5 and 95th percentiles of the simulated populations.

Visual predictive checks of the model in four animal species of the corresponding dose group were presented in **Figure 6**. In each check, most of the observed data located within the area between the 5 and 95th percentiles of the simulations, while the median of observed data approached the 50th percentile of the simulations, suggesting that the deviation between observed data and predicted profiles was reasonable and could be characterized by the simulated population. The virtual trial simulation plots validated that good predictions were achieved by our model for the four species.

### Predicted Pharmacokinetic Profile of DPT in Human

After developing and validating the PBPK model in the four preclinical species, we attempted to predict pharmacokinetic profile of DPT in human. *K*_t:pl_ values in human were also derived from rat based on the assumption that *K*_t:pl,u_ values were equivalent. Since no clinical information of DPT was reported, the dose for simulation in 70 kg human was assumed to be 16 mg according to the safety evaluation in monkeys, whose maximum tolerated dose was determined to be 4 mg/kg. The plasma concentration-time profile in human from 0 to 240 min after intravenous administration of 16 mg DPT was predicted (**Figure 4E**) and the pharmacokinetic parameters were estimated (**Table 3**). The predicted results showed that DPT exhibited a high *CL* of 1.39 L/min (close to hepatic blood flow) in human, with a *t*_1/2_ of 70.22 min and a *V*_ss_ close to total body volume (61.94 L).

### Sensitivity Analysis

**FIGURE 7:**
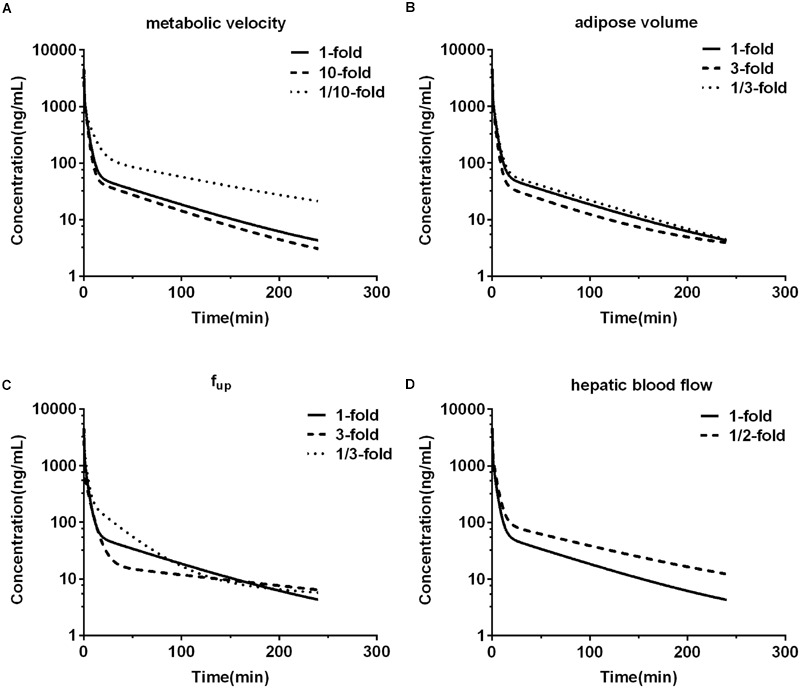
**The effects of changing metabolic velocity (A)**, adipose volume **(B)**, unbound fraction in plasma (*f*_up_) **(C),** and hepatic blood flow rate **(D)** in human PBPK model on predicted human plasma concentration-time profile of DPT.

Impacts of alterations in hepatic metabolic velocity of DPT, adipose volume, unbound fraction of DPT in plasma, and hepatic blood flow rate on the predicted pharmacokinetic behavior in human were documented (**Figure 7**). It was consistent with our expectation that metabolic velocity remarkably affected the exposure of DPT in human. When metabolic velocity was reduced to 1/10, AUC_0-∞_ increased to about 2.2-fold, accompanied by a fall in *CL* by 53.7%. However, when metabolic velocity was increased to 10-fold, AUC_0-∞_ only decreased to 86.2% of the original value, with an increase in *CL* of 16.0%. Tripled adipose tissue volume diminished AUC_0-∝_ by 20.1%, while raised *V*_ss_ by 43.7% and *t*_1/2_ by 40.0%. Slight variations in parameters were found when volume of adipose tissue was changed to 1/3 of the initial value. Unbound fraction of DPT in plasma prominently affected *t*_1/2_ and *V*_ss_. Threefold unbound fraction led to remarkable augments in *t*_1/2_ and *V*_ss_ to 224.1 and 246.7% of the control values, respectively, while AUC_0-∝_ and *CL* altered little. When unbound fraction in plasma was set to be 1/3 of initial value, *CL* decreased to 68.8% of the control, with *t*_1/2_ and AUC_0-∝_ rising by 123.2 and 45.3%, respectively, while *V*_ss_ slightly changed. Within our expectation, halved hepatic blood flow rate remarkably reduced *CL* to 61.6% and raised AUC_0-∝_ to 162.4% of the original data, respectively, displaying the characteristic of high hepatic extraction ratio drug.

### Interspecies Allometric Scaling

**FIGURE 8:**
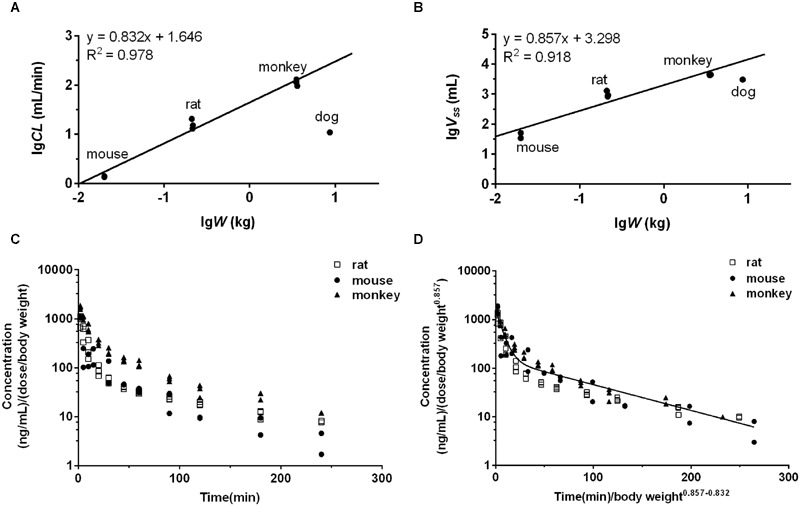
**The interspecies allometric scaling of DPT across mouse, rat and monkey and corresponding Dedrick plots.** The regression analyses of mean *CL* **(A)** and *V*_ss_ **(B)** of each dose group against body weight (*W*) were performed after log-log transformation across mouse, rat, and monkey. Dog’s data were not involved in the regression due to the low *CL* and *V*_ss_. The lines represent the regression lines across mouse, rat and monkey. **(C)** Mean plasma concentration normalized by dose (mg) per body weight (kg)-time profiles of DPT in mouse, rat, and monkey of each dose group. **(D)** Mean plasma concentration-time profiles of DPT in mouse, rat, and monkey of each dose group after transformation by Dedrick’s approach. The line denotes the pharmacokinetic curve simulated from the mixed profile.

The observed *CL* and *V*_ss_ of each species were incorporated to perform interspecies allometric scaling. It is generally accepted that *CL* (mL/min) and *V*_ss_ (L) increase with the rising body weight across species, whereas the parameters of DPT in dogs did not obey the rule. *CL* (10.94 mL/min) and *V*_ss_ (3.05 L) in dogs were lower than those in monkeys (*CL* 113.39 mL/min and *V*_ss_ 4.40 L, mean value of three dose groups), even though mean body weight of dogs (8.65 kg) was larger than that of monkeys (3.51 kg). Correction by unbound fraction of DPT in plasma might partly improve interspecies scaling of *V*_ss_, but did not improve scaling profile of *CL*. Neither maximum lifespan potential nor brain weight correction (Mahmood et al., 2006) substantially altered the scaling profile of *CL*. All these results demonstrated that dog seemed not to be suitable for interspecies scaling in the study. Therefore, interspecies scaling was carried out only across mouse, rat and monkey. Equations describing the relationship between the related parameters and body weight (*W*) across mouse, rat and monkey were *CL* = 44.28*W*^0.832^ and *V*_ss_ = 1.99*W*^0.857^, respectively (units: *CL*: mL/min; *V*_ss_: L; *W*: kg). Their correlations were fairly good (*r*^2^ > 0.91) (**Figures 8A,B**). The estimated human *CL* and *V*_ss_ based on allometric equation were 1.52 L/min and 75.65 L. Dedrick plot was generated across the three species, in which X axis was time divided by *W*^0.857-0.832^ and Y axis was concentration divided by (dose/*W*^0.857^). Plasma concentration-time data from mice, rats, and monkeys of all dose groups were used in the animal scale-up study. As seen in **Figures 8C,D**, the concentration-time profiles of DPT in three species are close to each other after being transformed. The mixed profile was further fitted to two-compartment model (resulted *C′* = 1.495e^-0.164^*^t′^*+ 0.156e^-0.012^*^t′^*, units: *C*′: μg/mL; *t*′: min). Plasma concentration-time profile following intravenous administration of 16 mg DPT in human (70 kg) was predicted by reverse transformation from the model above and a two-compartment model equation of *C* = 0.628e^-0.147^*^t^* + 0.066e^-0.011^*^t^* (units: *C*: μg/mL; *t*: min) was obtained (**Figure 4E**), from which pharmacokinetic parameters of human were estimated. The estimated AUC_0-∝_, *CL, V*_ss_, and *t*_1/2_ from Dedrick plot were 10.25 μg⋅min/mL, 1.56 L/min, 87.67 L and 63.08 min, respectively, which were close to the results by PBPK model.

## Discussion

As a potential anti-tumor candidate, DPT is undergoing preclinical evaluation up to date. Mouse, rat, monkey and dog are the most frequently used preclinical species. In this study a PBPK model was established to predict pharmacokinetic behaviors of DPT in all the four species above. The validated model was further used to predict pharmacokinetics of DPT in human.

Although, PBPK model may complete the prediction based on parameters obtained from *in vitro* and *in silico* data, many cases of predictions were still dependent on animal experiments, which supplied necessary parameters such as *K*_t:pl_ and *CL* for simulation (Hudachek and Gustafson, 2013; Hao et al., 2014; Hu et al., 2014; Bi et al., 2016). Since one of the most important applications of PBPK model is to make prediction prior to *in vivo* experiments (especially clinical trials), qualified models relying on *in vitro* and *in silico* inputs will be appreciated. In the present study, the PBPK model incorporated parameters based on *in vitro* and *in silico* research and gave reasonable predictions in various preclinical species, indicating the application of the model in the human pharmacokinetics projection.

Several *in silico* methods to estimate *K*_t:pl_ have been reported (Poulin and Theil, 2002; Berezhkovskiy, 2004; Rodgers and Rowland, 2006; Schmitt, 2008; Peyret et al., 2010; Ruark et al., 2014). In the present study, *K*_t:pl_ values in rat were calculated using the approach developed recently by Ruark et al. (2014), which was considered to provide a more comprehensive tissue composition model than previous approaches. In a previous attempt we employed *K*_t:pl_ values of rat throughout the models, resulting in poor predictions in the other three animals, which might be due to large interspecies difference in unbound fraction of DPT in plasma (from 5.39% in rat to 0.16% in dog). Correction of *K*_t:pl_ from rat by unbound fraction of DPT in plasma greatly improved the predictions in the other three species. Preliminary prediction showed an overestimation of DPT concentrations in brains of mice, which might be partly attributed to the restricted penetration of DPT through blood brain barrier. Introduction of the permeability-limited model into brain greatly improved the accuracy of prediction.

According to the previous study (Xie et al., 2016), DPT is mainly metabolized to M2 and M7 in liver microsomes of five species, both of which are further transformed to M1. Calculated by the data obtained in (Xie et al., 2016), the summed amount of M1, M2, and M7 formation counted for 84.7, 86.5, and 75.0% of DPT depletion in mouse, rat, and dog microsomes, respectively, after incubation of 60 min, while in monkey and human microsomes the data approached 100%. Thus, we assumed that DPT was eliminated through M2 and M7 formation in our PBPK model. The kinetics of M7 formation were studied and suggested auto-activation characteristics in microsomes of all species, which was diagnosed by the “hook” shapes in corresponding Eadie–Hofstee plots (Houston and Kenworthy, 2000). In human microsomes the sigmoidal kinetics of M7 was less obvious, with a relatively small γ value (1.22). Compared with M7, M2 formation was the major metabolic pathway of DPT in all species. It was seen from the estimated metabolic parameters (**Table 2**) that *CL*_M2_ was approximately at least fifty times larger than *CL*_M7_, indicating that the contribution of M7 formation to DPT elimination was minor.

Given the reasonable prediction in all four animal species, the developed PBPK model was further applied to characterize pharmacokinetics of DPT in human. The predicted *CL* (1.39 L/min) was close to hepatic blood flow rate, showing that DPT was subjected to high hepatic extraction in human. DPT was reported to be mainly metabolized to M2 in human hepatic microsomes, whose intrinsic clearance was up to 1.38 mL/min/mg protein (Xie et al., 2016). This might be a reason for the short elimination half-life (70.22 min) in human. The predicted *V*_ss_ was 61.94 L (close to total body volume), implying that DPT was extensively distributed in body.

Sensitivity analysis in human showed that the decrease in metabolic velocity increased exposure of DPT in plasma. In human, DPT is mainly metabolized by CYP2C9 and CYP2C19 (Xie et al., 2016), which exhibit extensive polymorphism in population (Hirota et al., 2013). In addition, DPT itself is a strong inhibitor of CYP2C9 (Lee et al., 2010). Therefore, high attention should be paid to the increased plasma exposure of DPT due to co-administration with CYP2C inhibitors, auto-inhibition of DPT on CYP2C9, and the administration in CYP2C poor metabolizers in the future clinical applications. DPT had high plasma protein affinity and variation of unbound fraction in plasma in population might affect DPT disposition. It was found that threefold unbound fraction led to an augment in *V*_ss_ by 146.7%, with AUC_0-∝_ changing slightly. However, one third of initial unbound fraction in plasma significantly decreased *CL* without affecting *V*_ss_, raising AUC_0-∝_ by 45.3% subsequently. It was in line with the characteristics of a high hepatic extraction ratio drug that *CL* of DPT in human highly depended on hepatic blood flow rate. Chemotherapeutical agents are able to induce liver injury (Ramadori and Cameron, 2010), leading to reductions in hepatic blood flow rate and plasma protein level. These alterations may greatly change pharmacokinetic behavior and affect safety and efficacy of DPT.

As a mechanical and dynamic approach PBPK model is believed more potent than interspecies allometric scaling in the pharmacokinetics prediction. In our study, although similar results were obtained by the two methods, interspecies allometric scaling exhibited high dependence on animal species. It was found that *CL* and *V*_ss_ of DPT in dogs were not fit for the scaling. Several efforts of correction failed, leading to a compromise to leave out dog’s data in the scaling. The disposition of DPT in dogs was greatly different from other species, with a very low clearance (less than 4% of hepatic blood flow) and low *V*_ss_ (35% of body volume), which might result from its low metabolic velocity in hepatic microsomes (Xie et al., 2016) and high plasma protein binding. These reasons explained why dog was not suitable in interspecies scaling to some extent. Whereas, PBPK model took the mechanical factors like metabolic velocity and unbound fraction in plasma into consideration and achieved acceptable predictions in dog, illustrating its extensive applicability in various species.

In addition, compared with the time- and animal-consuming approach of interspecies scaling, PBPK model achieves prediction more economically and efficiently solely from *in vitro* experiments along with literature data. It will contribute to screening the most promising candidates with ideal pharmacokinetic behaviors out of the substantial compounds. The mechanism-based model can also provide information in special populations such as children and hepatic insufficiency patients, as with the examples in sensitivity analysis.

In summary, a whole-body PBPK model of DPT in various species was developed mainly based on *in vitro* and *in silico* data. The predictions were validated by *in vivo* pharmacokinetic data from mice, rats, monkeys and dogs, along with visual predictive checks. The validated PBPK model as well as allometric interspecies scaling were tried to predict human pharmacokinetic profiles. We expect that the prediction will be valuable for dose selection and informative decision making during future clinical trials, and provide a reference for the PBPK studies of similar compounds.

## Author Contributions

YC, LL, XL, and ZZ participated in research design. YC, FL, QX, and MM conducted experiments. XL, YC, and KZ performed data analysis. YC, KZ, and LL wrote the manuscript.

## Conflict of Interest Statement

The authors declare that the research was conducted in the absence of any commercial or financial relationships that could be construed as a potential conflict of interest.

## Abbreviations

AUC_0-tn_: area under the plasma concentration-time curve from time zero to last time
AUC_0-∞_: area under the plasma concentration-time curve from time zero to infinity
*CL*: plasma clearance
DPT: deoxypodophyllotoxin
*f*_up_: unbound fraction in plasma
*K*_m_: Michaelis–Menten constant
*K*_t:pl_: tissue-to-plasma concentration ratio
*K*_t:pl,u_: unbound tissue-to-plasma concentration ratio
LC–MS/MS: liquid chromatography–tandem mass spectrometry
MRT: mean residence time
PBPK: physiologically based pharmacokinetic
*PBSF*: physiological-based scaling factor
*PS*: permeability-surface product
*t*_1/2_: terminal half-life
*V*_max_: the maximum metabolic velocity
*V*_ss_: apparent volume of distribution at steady state
*W*: body weight
